# An Effective Filter for IBD Detection in Large Data Sets

**DOI:** 10.1371/journal.pone.0092713

**Published:** 2014-03-25

**Authors:** Lin Huang, Sivan Bercovici, Jesse M. Rodriguez, Serafim Batzoglou

**Affiliations:** Department of Computer Science, Stanford University, Stanford, California, United States of America; Swiss Federal Institute of Technology (ETH Zurich), Switzerland

## Abstract

Identity by descent (IBD) inference is the task of computationally detecting genomic segments that are shared between individuals by means of common familial descent. Accurate IBD detection plays an important role in various genomic studies, ranging from mapping disease genes to exploring ancient population histories. The majority of recent work in the field has focused on improving the accuracy of inference, targeting shorter genomic segments that originate from a more ancient common ancestor. The accuracy of these methods, however, is achieved at the expense of high computational cost, resulting in a prohibitively long running time when applied to large cohorts. To enable the study of large cohorts, we introduce SpeeDB, a method that facilitates fast IBD detection in large unphased genotype data sets. Given a target individual and a database of individuals that potentially share IBD segments with the target, SpeeDB applies an efficient opposite-homozygous filter, which excludes chromosomal segments from the database that are highly unlikely to be IBD with the corresponding segments from the target individual. The remaining segments can then be evaluated by any IBD detection method of choice. When examining simulated individuals sharing 4 cM IBD regions, SpeeDB filtered out 99.5% of genomic regions from consideration while retaining 99% of the true IBD segments. Applying the SpeeDB filter prior to detecting IBD in simulated fourth cousins resulted in an overall running time that was 10,000x faster than inferring IBD without the filter and retained 99% of the true IBD segments in the output.

## Introduction

Identity by descent (IBD) is a fundamental concept in genetics, pertaining to the genetic similarities among individuals who co-inherited an allele from a common ancestor. While genomic sequence variants, such as single-nucleotide variations, insertions, deletions, and copy-number variations, are constantly being introduced with every generation, genomic IBD segments have a high probability of being identical due to the relatively low de-novo mutation rates [1], [2]. We call a sequence of alleles IBD if the alleles have been inherited from a common ancestor without recombination events. Because relatively few recombination events occur in each generation (roughly one per chromosome), the two likely-related individuals co-inherit a genomic segment decreases rapidly with every generation, while IBD segments typically stretch to significant lengths. Specifically, in the case of two relatives that share a relative 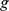 generations ago, a genomic segment that originated from the common ancestors needs to be transmitted over 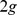 meioses, which corresponds to a probability of 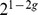. Once transmitted, assuming recombinations follow a Poisson process [3], the expected length of the shared segment is 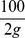 centimorgans (cM).

Single-nucleotide polymorphisms (SNPs) are commonly used to determine whether genomic regions are IBD. Opposite homozygous loci [4] are positions where two individuals are homozygous for different alleles. Within co-inherited regions, two genotyped individuals will not exhibit opposite homozygous loci, except in case of genotyping errors or mutations that arose more recently than the common ancestor. This simple observation can be the basis of an effective IBD filter, as we demonstrate in this article.

Many biological applications are based on our ability to determine whether or not two individuals inherited a genomic region from a single ancestor [5], [6]. Researchers exploring ancient population histories rely on the ability to trace population origin and admixture dynamics through the accurate detection of shared segments [4]. Studies that map disease genes rely heavily on the ability to find genomic regions shared by cases. For example, in association studies, one must control for spontaneous sharing, while enriched sharing near a particular locus can be indicative of a disease association gene [7].

Extensive previous work has focused on IBD inference, aiming at increasingly longer time-scales, ranging from recent familial relatedness, up to several tens of generation to the most recent common ancestor.

PLINK [6] analyzes pairs of individuals using a simple three-state model HMM. The states in the model correspond to the amount of shared IBD per position given the observed genotypes of two individuals. BEAGLE [8] uses a factorial HMM to phase the individuals’ genotypes and then determines shared haplotypes between individuals. Unlike Plink, the BEAGLE model incorporates complex linkage-disequilibrium (LD) patterns by constructing a compact set of states and constrained transition probability matrix that are equivalent to a variable-length Markov model. More explicit modeling of the inheritance vector, capturing the relationship between individuals, is described in earlier work that incorporated LD via a first-order Markov model at the level of the founders [5]. This explicit modeling of both relationship and LD is shown to significantly improve performance. In the work by Moltke et al., a Markov Chain Monte Carlo (MCMC) approach for IBD inference is presented whereby segments of chromosomes are iteratively partitioned into sets of identical descent [9]. The above methods present a tradeoff between accuracy and running time, where more complex methods require longer running time for the analysis. More importantly, the complexity of the analysis in all these methods is quadratic in the number of individuals, as every pair of individuals is evaluated. GERMLINE [10] reduces the time complexity of IBD inference at the cost of lower accuracy. The key idea is to perform the analysis on phased data; by employing hash tables with segments taken from the phased data, the method efficiently identifies seeds of segments that potentially represent IBD segments shared between individuals. Henn et al. [4] infer IBD from unphased genotype data by comparing SNPs of two individuals and identifying opposite homozygous SNPs. A region is inferred as a half IBD if (i) two individuals do not have opposite homozygous SNPs, (ii) the region is no shorter than 5 cM, and (iii) at least one of these two individuals has more than 400 homozygous genotyped SNPs in this region. The work addresses genotyping errors by heuristically allowing one opposite homozygous SNPs assuming a region is at least 3 cM and it has at least 300 SNPs. While the method is highly efficient, in practice, it can be applied for cases where the expected IBD segments are longer than 7 cM, or otherwise the method exhibits low sensitivity. IBD-Groupon [11] constructs an HMM model to detect IBD segments shared by multiple individuals. Although this method is able to detect group-wise IBD segments among hundreds of individuals sampled over thousands of SNPs, it relies on pairwise IBD inference using BEAGLE. PARENTE [12] detects related pairs of individuals accurately and rapidly from unphased genotyping data using an embedded likelihood ratio test. Similar to many other previous work, this method examines every pair of individuals.

As studies grow to include large cohorts and meta-analyses include many diverse data sets, the accuracy of IBD inference methods becomes extremely important. However, the running time of these accurate IBD inference methods makes them computationally infeasible to apply on very large data sets. Here, we describe an efficient and accurate filtering method for IBD detection which we named SpeeDB. Given a query unphased genotyped individual and a database of unphased genotyped individuals, SpeeDB rapidly screens out genomic regions in the database that are unlikely to be IBD with the query, and returns the remainder of genomic regions. These regions can be passed onto traditional IBD inference methods such as PARENTE [12], fastIBD [13], or GERMLINE. Our method is designed so as to readily translate into an indexing scheme that can be used for searching for IBD segments within a large database of individuals. Along with the developed fast query approach, our method thus provides a practical infrastructure for large-scale IBD detection.

IBD inference methods developed so far have focused on applications where the analysis is performed once on a collected cohort. With the exponential increase in GWAS studies, meta-analysis further gained momentum, merging samples from several studies in an attempt to leverage the large data sets to increase the statistical power. We developed SpeeDB, in part, to streamline such analyses and provide a continuous solution to the problem of IBD inference. We chose to architect our method so as to allow the continuous addition of samples to an on-going study; newly sampled individuals are queried against a growing database of samples for shared ancestry.

## Methods

**Figure 1:**
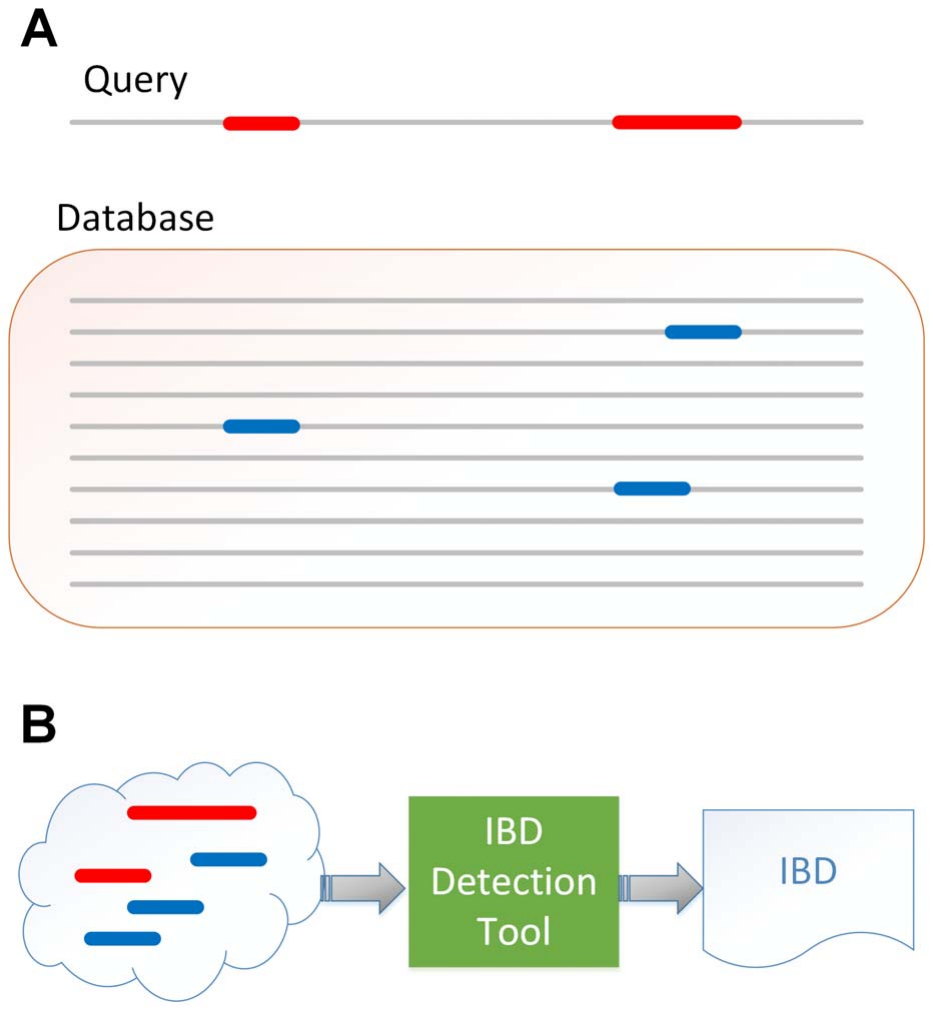
Overview of SpeeDB and its downstream application. (A) SpeeDB identifies a set of candidate genomic segments (blue) of individuals in a database and the corresponding genomic segments of a query individual (red) that may be IBD. (B) Candidate segments from the database and the corresponding regions in the query will be sent to an accurate IBD detection tool (such as PARENTE [12]) for inference. Thus, SpeeDB greatly reduces the time necessary to infer IBD on large data sets by reducing the total amount of data on which inference must be applied.

IBD inference is commonly applied on a set of individuals. Toward the goal of identifying IBD between all pairs of individuals in a cohort, we focus on a first step of identifying potential IBD between a single individual and members of a cohort (see Fig. 1). For the description of our method, we borrow the terms *query* and *index* from the database nomenclature to refer to the given individual (the *query* individual), and the other members of the cohort (the *indexed* individuals).

We first assume that individuals are measured with no genotyping errors at the sampled markers. The above assumption will be relaxed in the next section. Our method relies on the observation that given no genotyping error, two related individuals should have at least one allele in common, resulting in no opposite homozygous SNP sites along the co-inherited segment. Conversely, when opposite homozygous SNPs are observed, under the assumption of no genotyping errors, two individual cannot be related in the corresponding site. Note that, we do not account for the rare event of de novo mutation for ease of discussion. It follows that given the set of homozygous minor allele sites in the query individual, indexed individuals can be scanned along these markers; indexed individuals exhibiting homozygous major allele calls at any of these positions can be discarded from further consideration around that particular marker location.

**Figure 2:**
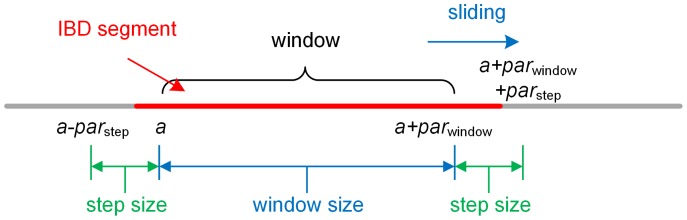
Sliding window along a chromosome. When processing a query genome, we consider a window with size 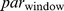 cM in each step, where 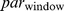 is less than the target IBD block length 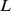. The indexed individuals that do not exhibit opposite homozygous SNPs with respect to the query in this window define the set of candidate individuals that may be IBD with the query around this window. In the subsequent iteration, the window moves along the chromosome by 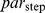.

Our method consists of two steps: indexing and query processing. First, during the *indexing* step, individuals’ genotypes are scanned and transferred so as to allow efficient query. In this step, for every marker 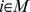 of the 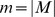 biallelic markers, we generate a set of indexed individuals 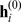, in which every element is an indexed individual that is homozygous in the major allele. Given a database with 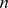 individuals sampled over 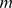 biallelic markers, this step takes 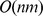 time. The computation is performed once, and the resulting sets are stored on disk. During the query phase, given a query individual, we use a sliding window 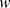 along the corresponding query genome. The set of markers located within a window 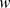 are denoted by 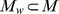. The sliding window process is parameterized by the window size 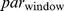 and step size 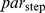. As we slide the window along the genome, we narrow our search to specific markers where the query individual has homozygous minor alleles, denoted as 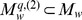. For every such position, we examine all selected markers within the current window; we identify all individuals that do not appear in any 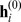 sets, for any 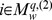 where the query individual 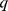 is homozygous minor within the region indicated by the window 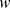. These individuals potentially share IBD segments with the query individual, within regions that harbor the examined window. As such, the size of the window 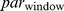 constitutes a lower bound for the potential IBD segment length. As one end of the IBD segment can potentially lie within 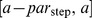, and the other lies within 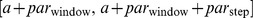, we report 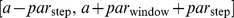 as the candidate segment. Intersecting candidate segments produced by subsequent windows analysis are merged, producing the final reported set of candidate regions. When examining a window, the set 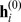 is visited exactly once for all 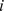 where the query individual is homozygous in the minor allele, hence the expected computation of a query processing is 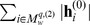. Let 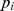 be the minor allele frequency (MAF) at marker 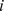. Given the expected set size of 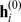 can be estimated as 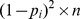 and that the probability of the query individual exhibiting homozygous minor at marker 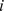 (where 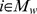) is 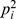, the expected number of operations is given by 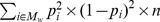. Once a window has been examined, the analysis advances to the next examined window 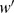, by 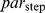 cM. Rather than performing all of the above calculations from scratch for each window, we reuse many of the calculations in the previous window when we slide to the next one by eliminating the impact of 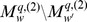 and add the impact of 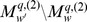. This process is repeated for the entire genome. The process is shown Fig. 2, illustrating how the IBD segment, marked in red, is targeted for detection.

To further increase the efficiency by which we prune the candidate list, a second filter is applied focusing on positions where the query individual is homozygous in the major allele can be derived. Using this second filter, one can eliminate segments corresponding to indexed individual that is homozygous in the minor allele. An indexing phase, similar to the one described above, is performed for this second filter prior to query. A second set 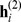 is computed for every marker 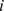, containing indexed individuals that are homozygous in the minor allele at this marker. We refer to the first and second described filters as the *Major Filter* and the *Minor Filter* throughout the text, respectively. The output of SpeeDB are segments that pass both filters.

### Tolerating Genotyping Errors

In the above section, we assumed that no genotyping errors are present in our samples. The assumption significantly simplified calculations; however, the reality is that every sample will have many genotyping errors. Genotyping errors can introduce false-positive IBD candidates, as well as falsely remove true IBD segments from the candidates set. New false-positive segments may be introduced in cases where opposite homozygous calls in either the query or the indexed individuals have been disrupted by the error. Alternatively, a true candidate may be falsely removed from the list in cases where opposite homozygous calls were introduced by errors. As our method’s main objective is to provide a filtering process achieving sufficiently high sensitivity (i.e., a method that does not eliminate true positives), we chose to optimize on the latter. Namely, our method will only account for genotyping error that might remove a true IBD candidate from the list.

Let 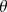 be the probability of a genotyping error occurring, defined as the probability that an allele in the genotype call is incorrect. We denote the minor allele count at position 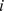 as genotype 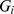. Table 1 lists the probabilities for an observed minor allele count, given the true underlying allele count.

**Table 1:** The probability of the observed genotypes 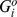 given the true underlying genotype 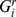.

True genotype 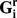	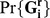	Observed genotype 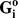	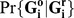
2	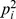	2	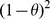
		1	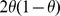
		0	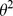
1	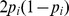	2	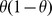
		1	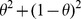
		0	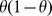
0	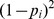	2	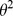
		1	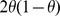
		0	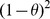

Given the true genotype at a marker 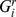, the conditional probability of observing an genotype 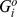 can be written as a function of the error rate 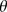.

Consider all the selected markers within a window. One can accurately compute the probability of the observed opposite homozygous SNPs between the query and an indexed individual while accounting for genotyping errors. If the observed opposite homozygous SNPs have a sufficiently high probability of being the result of genotyping errors given the other evidence that support the presence of an IBD segment, the filter should not screen the corresponding segment.

However, the exact computation of this probability can be relatively time-consuming, especially in the case of large data sets and those with high SNP density. To reduce computation time, we approximate the probability that the query has 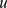 false opposite homozygous SNPs within the examined window with respect to any indexed individual, denoted by 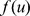. Given this approximated probability, we find the minimum value 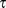 that guarantees that the probability of having more than 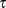 false opposite homozygous calls is bounded by some pre-defined low probability. In the database processing, we count the number of the observed opposite homozygous SNPs in each indexed individual, and eliminate the ones with more than 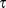 observed opposite homozygous SNPs, as these individuals are deemed as likely to have true opposite homozygous calls with the query.

Consider the *Major Filter* first. Given the query individual, we are able to predict the probability of having a false opposite homozygous call at a certain position with respect to an indexed individual. By definition, an opposite homozygous SNP is observed at a certain position if the following two statements are both true: (i) the marker was selected during marker filter construction, and (ii) the indexed individual has an observed major allele count of 2 at the same marker. Let 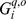 and 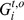 correspond to the observed minor allele count at position 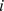 for the query individual 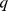 and an indexed individual 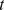, respectively. Similarly, let 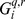 and 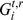 correspond to the true underlying minor allele count at position 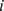 for the query individual 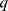 and the indexed individual 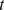, respectively. The above two statements can be represented as (i) 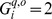 and (ii) 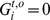. However, the underlying true minor allele count of the query could be either 0, 1, or 2, regardless of the observed homozygous minor allele measurement. The true genotype of an indexed individual could be different from the observation as well. These three cases are discussed below.

In the first case, the query truly has two copies of the minor allele, i.e., 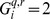. In this case, the genomic segment of the indexed individual should not be filtered out if it has at least one copy of minor allele. The probability of an indexed individual having a false opposite homozygous SNP in a selected marker where the query is truly homozygous minor is given by
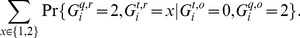
(1)


In the second case, the query is heterozygous, namely, 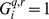. In this case, all the opposite homozygous SNPs at this marker will falsely generate a rejection. The probability for the event is given by
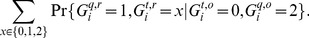
(2)


Finally, in the last case, the query has two copies of major allele (i.e., 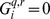). The genomic segments of indexed individuals with at least one copy of major allele should not be removed from candidate list. The probability for this event is given by
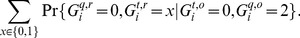
(3)


Summing up Eq. (1)-(3) gives us the probability of having a false opposite homozygous SNP between an indexed individual and the query in a certain marker position, conditioned on the fact that the marker meets the filter selection criteria. Assuming the query genotypes are independent, for any given marker 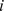, the probably of having a false opposite homozygous call due to genotyping error is given by the following summation:
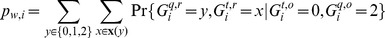
(4)where,



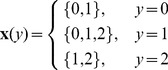
(5)The conditional probabilities of the observed genotypes given the true underlying genotypes are given in Table 1. Substituting these probabilities into Eq. (4) formulates 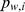 as a function of the genotyping error rate 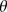 and the minor allele frequency 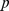.

The approximation of 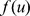 is specified below. We use a fixed value for the minor allele frequency 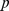 to represent all markers and compute 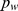 by substituting this value into Eq. (4). Then, assuming 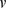 markers are selected in the current window, we estimate the probability that an indexed individual has 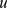 genotyping-error-induced false opposite homozygous SNPs by 
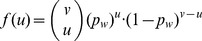
. Thus, to account for 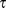 erroneous opposite homozygous SNPs, the probability that a genomic segment of an indexed individual being IBD with the query and will not pass the filter due to genotyping errors is estimated to be 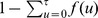. To bound it by a pre-defined threshold 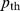, we find the minimum value of 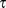 that guarantees that
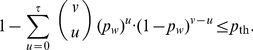
(6)


The value 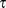 is positively correlated with the marker count 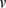 and is negatively correlated with the minor allele frequency 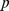. We conservatively set the fixed minor allele frequency 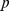 to be its minimum value observed in the window. By doing so, the filter maximizes tolerance with respect to false opposite homozygous SNPs.

When considering the *Minor Filter*, 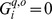 and 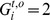 are considered as the opposite homozygous SNPs. Following similar argument as above, the conditional probability of having a false opposite homozygous SNPs at a certain marker location given the marker is selected for constructing the *Minor Filter* can be written as follows
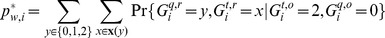
(7)where 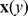 is as same as Eq. (5).

Similar to the analysis of the *Major Filter*, assuming 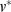 markers are selected, we need to find the minimum 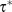 to guarantee
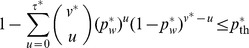
(8)where, 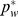 is computed with a fixed 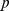 value. For this filter, we set the fixed 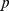 value to be its maximum value to be conservative.

### Improving Pruning Efficiency

As we perform that analysis on a dense set of bi-allelic markers, high levels of linkage disequilibrium are expected between neighboring SNPs. The non-random association between alleles can in turn result in high concordance between 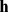 representing neighboring positions. Indeed, our analysis reveals that for neighboring markers present such high concordance. We can thus select only a subset of the markers; a single marker with set 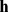 can represent other markers with the similar 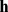
’s which will otherwise add a non-significant pruning power. Based on this observation, we use the following Jaccard index criteria:
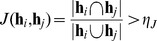
(9)where 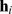 and 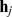 represent indexed individual sets for markers 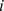 and 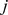, respectively. During the *indexing* phase, two markers with a Jaccard index higher than 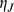 will be marked as representing approximately the same set using a unique label. During the *query* phase, when constructing the appropriate filter, only markers with unique labels are selected. Thus, the markers that will presumably add little pruning power are discarded.

## Results

### Experimental Setup

To evaluate the performance of SpeeDB, we used data from 255 individuals from Asian populations of the HapMap Phase III panel [14], and from 1,480 individuals from the 1958 Birth Cohort of the Wellcome Trust Case Control Consortium (WTCCC) [15]. Our analysis focused on the long arm of chromosome one which contained 36,638 and 13,943 SNPs for HapMap and WTCCC, respectively. We defined a set of indexed individuals from each data set using the genotypes of all individuals in the data set, which we call *HapMap* and *WTCCC*. We generated two additional data sets, *HapMap.lowD* and *HapMap.1M*, from HapMap by using all HapMap individuals but downsampling the markers used. *HapMap.lowD* was sampled to have the same marker density as *WTCCC*, and *HapMap.1M* was sampled to have a marker density corresponding to 1 million markers across the entire genome. We also combined the data from 14,318 individuals across all of the disease and control cohorts of the WTCCC study into a data set which we call *WTCCC.largeSet*. Since haplotypes were necessary for our simulations, we used only trio-based phased data from HapMap and we phased the WTCCC data using HAPI-UR [16].

For each data set, we simulated query individuals that each contained one shared 4 cM IBD segment (except where otherwise specified) with exactly one haplotype of one individual in the data set. To do this, for each query, we first created two *composite* haplotypes using the original haplotypes from the data sets in such a way to break up any latent IBD segments that may exist between individuals in the data set in a way that is similar to the approaches taken in previous work [12], [13]. Specifically, we first partitioned the genome into a series of 0.2 cM segments. Each segment of a composite haplotype was made by copying alleles from a randomly selected original haplotype as illustrated in Fig. S1A. Next, we simulated a 4 cM IBD segment between the query individual and a random indexed individual at a random location by copying one of the haplotypes of the indexed individuals over one of the haplotypes of the query individual in the segment boundaries (see Fig. S1B). Finally, we introduced errors in the query individual’s haplotypes with an error rate 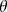. For each experiment, we generated a set of 10,000 queries.

Except where otherwise noted, the filter parameters were set as follows: 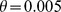 [12]; the window size 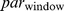 = 3.5 cM and the step size 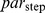 = 0.5 cM (appropriate for identifying 4 cM IBD segments); and the Jaccard index parameter 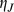 = 0.9. All experiments were performed on an AMD Opteron 6172 Processor.

### Experimental Results

We performed a number of simulations to demonstrate the efficiency and performance of SpeeDB on various data sets. To measure efficiency, we report the average *running time* for filtering a database based on a given query. To measure performance, we report the *sensitivity* as the fraction of IBD segments that pass SpeeDB’s filters and the *speedup* resulting from filtering out large portions of the database. The speedup is calculated as 1/(*candidate fraction*) where the *candidate fraction* is defined as the fraction of the database that passes the filters, where the database size is defined as the genetic length times the number of individuals in the database. That is, the candidate fraction represents the fraction of database that SpeeDB determines may have IBD segments with the query, which will then be passed on to a downstream accurate IBD detection method.

In the *HapMap* data set, SpeeDB was able to reduce the total amount of work required for downstream IBD detection by 99.5% while retaining 99% of the true IBD segments in the output and only took 3.2 ms per query to run. This effectively means that IBD can be detected 200 times faster while only reducing sensitivity by at most 1%.

We applied SpeeDB in conjunction with a state-of-the-art IBD inference method PARENTE and theoretically estimated the total running time necessary to run SpeeDB and then run PARENTE on the portion of the data that passed SpeeDB’s filtering. Our results in Table 2 show that SpeeDB’s running time is negligible compared to PARENTE, and that running PARENTE on the output of SpeeDB requires significantly less time than running PARENTE on the entire data set. We also compared the conjunction of SpeeDB and PARENTE’s running time to GERMLINE and fastIBD; the results are shown in the same table. Note that, SpeeDB and PARENTE are designed to operate on unphased genotype data. While GERMLINE can also run on unphased data, its accuracy in this mode is significantly lower, making it impractical to use on large data sets. We therefore ran GERMLINE on phased data, which consequently requires a phasing pipeline to be run prior to IBD inference. The running time in Table 2 includes this overhead.

**Table 2:** Running time.

	Time (sec)
SpeeDB	0.1
SpeeDB+PARENTE	2.2
PARENTE	427.0
GERMLINE	2,206.9
fastIBD	4,267.0

SpeeDB’s running time is shown in comparison to the running times of IBD detection methods PARENTE, GERMLINE, and fastIBD. SpeeDB+PARENTE denotes the total time for using SpeeDB followed by running PARENTE only on the candidate segments output by SpeeDB. Calculations based on analyzing 6,400 pairs of individuals (one query vs. 6,400 indexed individuals for SpeeDB and SpeeDB+PARENTE). SpeeDB was run with thresholds corresponding to 99% sensitivity.

**Figure 3:**
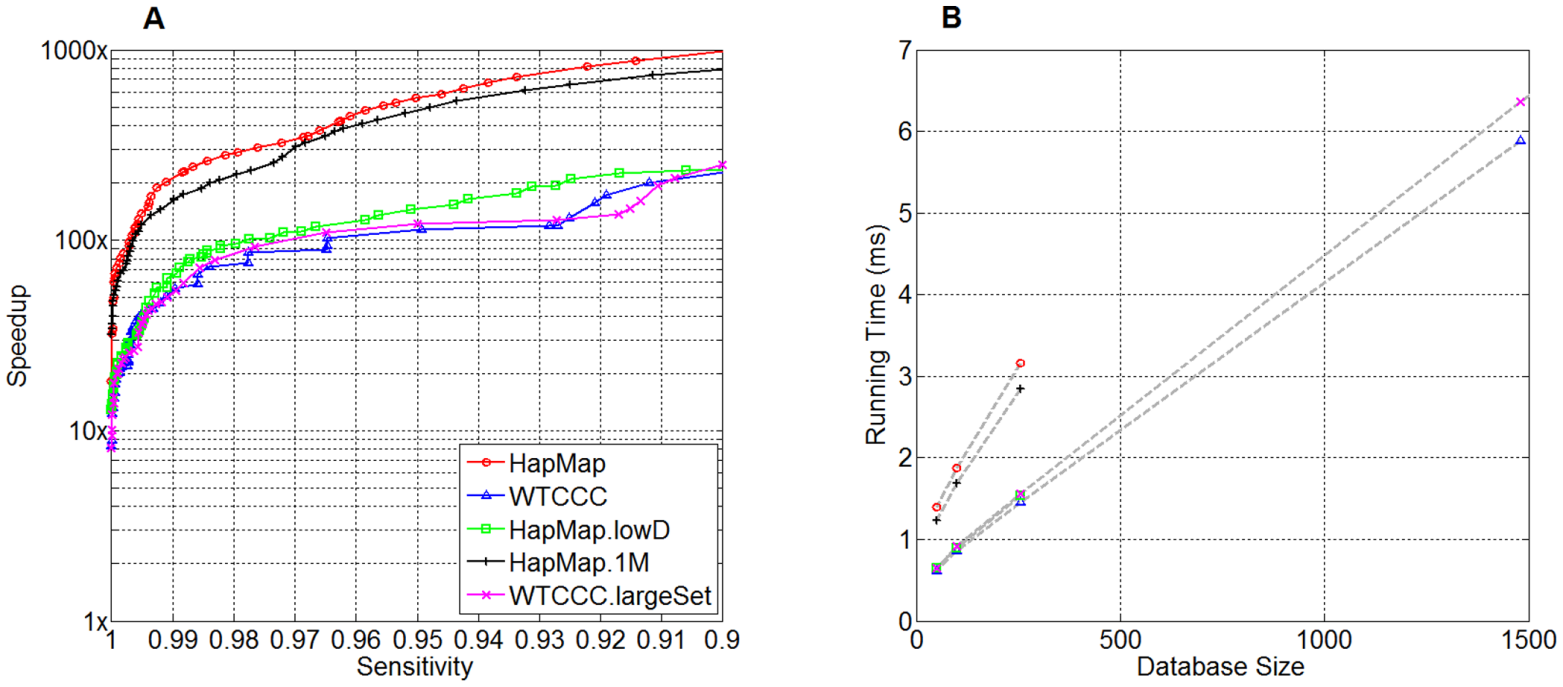
Performance of SpeeDB for identifying 4 cM candidate IBD segments. (A) The speedup achieved as a function of sensitivity. Speedup is measured as 1 divided by the fraction of the database retained after applying SpeeDB. Sensitivity is measured as the fraction of simulated IBD segments that pass SpeeDB’s filters. (B) The running time for comparing one query to the entire database.

The results of running SpeeDB on each data set are shown in Fig. 3. SpeeDB’s performance, measured by the speedup achieved by using the filter (assuming negligible filter running time) while retaining a particular sensitivity level, are shown in Fig. 3A. Each performance curve was generated by varying the error tolerance thresholds 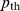 and 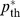 in Eq. (6) and Eq. (8). Because we had two thresholds to vary, we could not describe the performance of SpeeDB with a single ROC curve. Instead, it could be described with many potentially-overlapping ROC curves, each produced by fixing one threshold to a particular value and varying the other (see Fig. S2). For clarity, each performance curve in Fig. 3 shows the Pareto frontier (i.e. the leftmost points) of all the ROC curves generated for each data set. Because we show this frontier, there are a few bumps in the curves resulting in where the individual ROC curves intersect (see Fig. S2 to view the individual ROC curves for the *HapMap* data set shown here). It is interesting to note that the vast majority of the results with 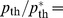 2.5 to 5 fall on or very close to the Pareto frontier in all the data sets evaluated.

The results in Fig. 3A show that SpeeDB filters out a large fraction of the database while maintaining a high level of sensitivity. For example, for the *HapMap* data set, it can attain a 99% sensitivity level while increasing the speed of IBD detection 200-fold by filtering out the 99.5% of the database as possible IBD locations. This figure also shows that SpeeDB performs better with higher marker density. We first observed that SpeeDB performs much better on *HapMap* compared to the WTCCC data sets and we also noted *HapMap* has higher marker density than the WTCCC data sets, so we suspected that this may be the reason. To confirm that marker density was a major contributing factor to better performance, we downsampled markers in *HapMap* to create *HapMap.lowD* to have the same the marker density as seen in *WTCCC*; 22,695 markers were removed randomly in this process. We then observed that the performance of *HapMap.lowD* is very similar to the performance of *WTCCC* and *WTCCC.largeSet*, indicating that marker density is a key factor affecting performance. Despite the lower density of *WTCCC*, SpeeDB still achieves a 54.3x speedup at 99% sensitivity on the *WTCCC* data set.

The efficiency of SpeeDB is illustrated in Fig. 3B and Table 2. These results show that SpeeDB only requires a few milliseconds to analyze each query.

To examine the scalability of SpeeDB, we created smaller databases by using subsets of individuals in the original databases. We observed that querying *HapMap* requires more time than querying *WTCCC* with the same number of individuals. We attributed it to the difference in marker density of these two databases. To illustrate this point, we used the *HapMap.lowD* data set. As shown in Fig. 3B, the running time for querying *HapMap.lowD* and *WTCCC* is almost identical. We also show that the running time is linear to the database size, implying that this tool can be utilized on even larger data sets. Its applicability to the large WTCCC data set with 14,318 individuals is demonstrated in Fig. S3. Assuming the marker density is as same as *WTCCC*, it is estimated that querying a database with 1 million individuals for IBD on the long arm of chromosome 1 takes roughly 4 seconds; assuming the marker density of *HapMap*, the estimated time is roughly 12 seconds.

**Figure 4:**
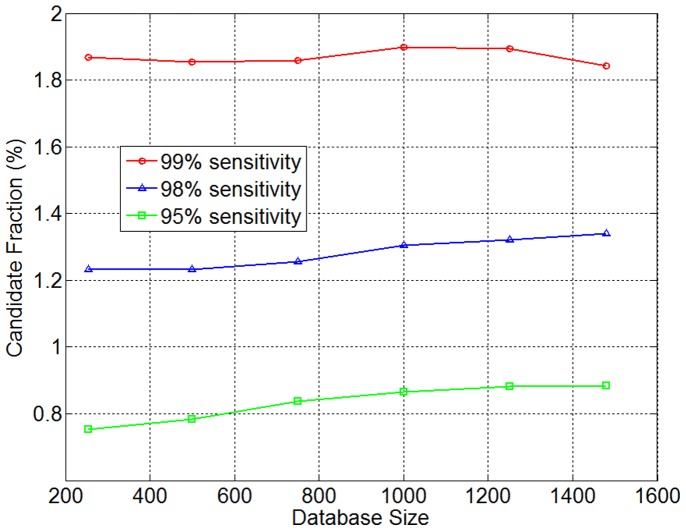
Fraction of database retained as a function of database size. The candidate fraction (the fraction of the database retained after applying SpeeDB) is observed to be nearly independent of database size at a given sensitivity level.

The scalability of SpeeDB in terms of pruning power is demonstrated in Fig. 4. Again, we created smaller databases by using subsets of individuals in *WTCCC*, and applied SpeeDB on these databases. Fig. 4 shows the candidate fraction as a function of database size at three sensitivity levels. For 99% sensitivity, the candidate fraction appears to be independent of database size, implying that the applicability of SpeeDB on large databases is promising.

We also investigated how often SpeeDB’s output contains the entire true IBD segment such that no part of the true IBD segment lies outside of the candidate regions. To do this, we computed the performance with the more stringent requirement that an IBD segment must be entirely covered by candidate regions to be considered correct, otherwise the part of the candidate region that overlaps the IBD segment are considered incorrect. In our experiments, we found that in the vast majority of cases, SpeeDB’s output correctly captures the entire true IBD region. For example, in the *HapMap* and *WTCCC* data sets, when SpeeDB identifies 99% of all markers in IBD (overall sensitivity), SpeeDB detects 98.76% and 98.65% of all IBD segments to be fully covered by candidate regions, respectively.

## Discussion

**Figure 5:**
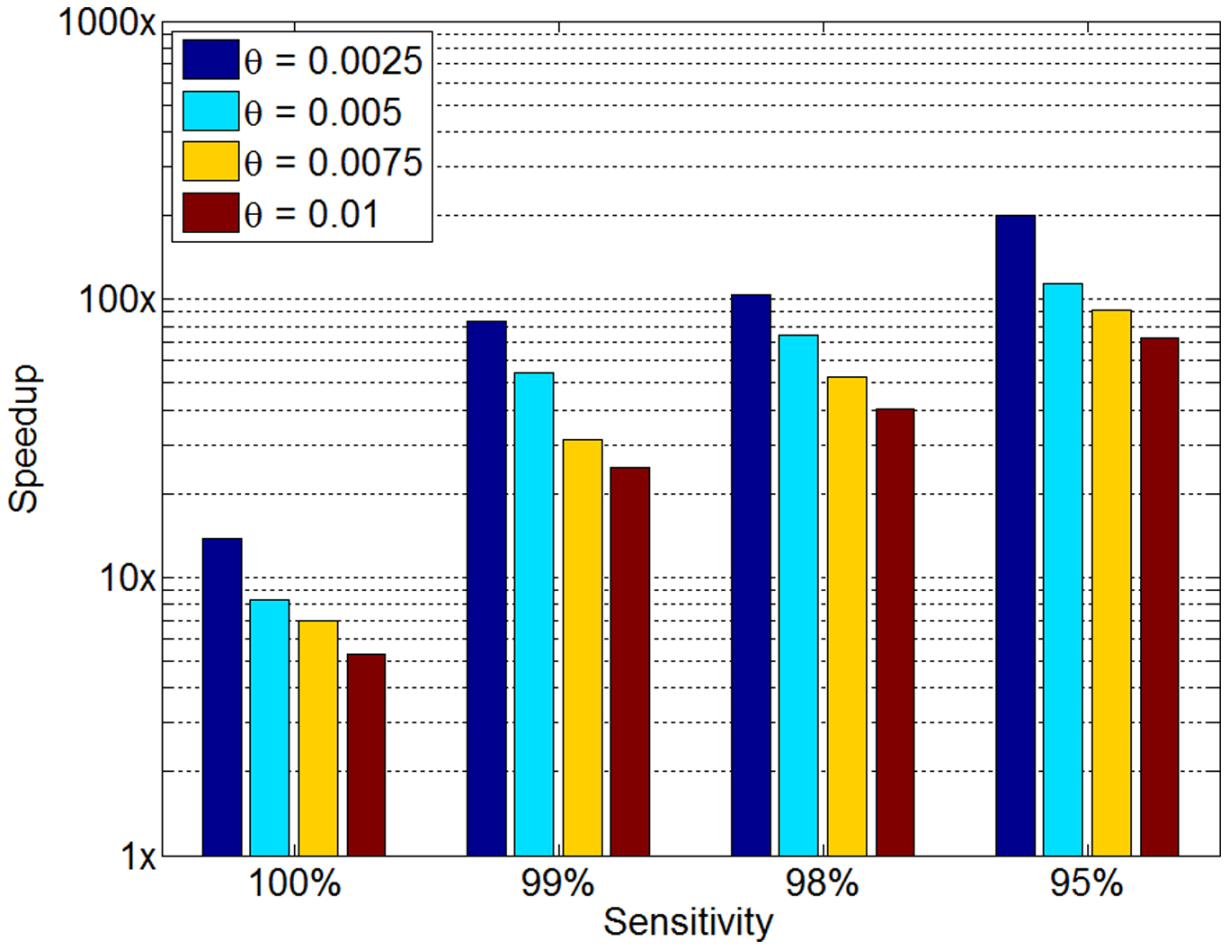
The performance of SpeeDB at various simulated error rates. Queries for the *WTCCC* data set were simulated with various error rates 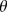 and the speedup observed remained within an order of magnitude even for high error rates.

In order to examine SpeeDB’s resistance to noise in the genotyping process, we ran several experiments varying the rate of simulated genotyping errors while fixing the modeled error rate 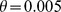 (therefore, we used the same 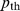 and 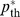). The results of this experiment are shown in Fig. 5 which demonstrates SpeeDB’s robustness to genotyping errors. At the 99% sensitivity level, when varying the error rate from 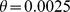 (a realistic error rate for modern genotyping arrays) to 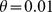, the speedup achieved remains within an order of magnitude, between 25x and 84x.

**Figure 6:**
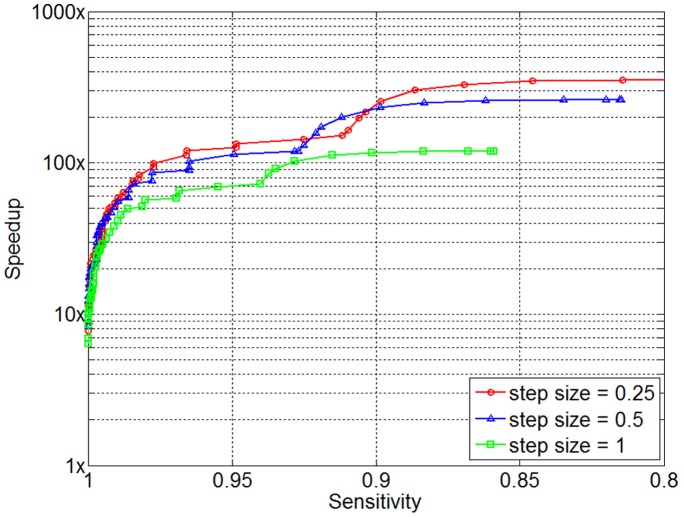
The influence of parameter *par*_step_. SpeeDB was applied on the *WTCCC* data set with various step sizes. While lower step sizes results in higher accuracy, it comes at the cost of more computational time.

The choice of step size also influences the performance of SpeeDB; a larger step size results less computation but reduced effectiveness. This is because at a particular window, SpeeDB reports on the potential IBD status the window itself plus the 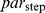 cM flanking both sides of the window. Because 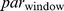 was set to the injected IBD segment size minus 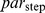, increasing the step size reduces the expected number of markers that will be used to perform filtering, resulting in reduced effectiveness. However, a larger step size means fewer windows will be visited overall, resulting in fewer computations. We ran SpeeDB with various step sizes in order to measure its accuracy as a function of step size and the results are shown in Fig. 6. With the step size increasing from 0.25 cM to 0.5 cM to 1 cM, the running time per query decreases from to 7.6 ms to 5.9 ms to 5.5 ms, but the speedup observed at 99% sensitivity decreases from 55.1x to 54.3x to 40.2x.

SpeeDB is composed of two filters. When the minor allele frequency is very low, we noticed that the *Major Filter* is very sensitive to the genotyping errors. With this observation, we eliminated low MAF (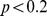) markers from this filter. Table 3 shows that each filter by itself has comparable pruning power, however the combination of the two yields significantly higher performance. Additionally, the running time on these two filters are roughly equal.

**Table 3:** The contributions of the *Major Filter* and the *Minor Filter* to the overall performance.

	Speedup		
Sensitivity	Both filters	*Major Filter* only	*Minor Filter* only
99%	54.3x	10.4x	9.3x
98%	74.6x	13.7x	10.9x
95%	113.2x	18.6x	17.4x

Each filter has approximately the same pruning power and using both together results in the best performance.

We introduced an optimization technique in the Methods section to ignore markers that provide little pruning power for a given query. Intuitively, when most of the indexed individuals have the same genotypes at two markers, using both markers for filtering provides little additional filtering benefit over using just one of them. Therefore, we applied the Jaccard index criteria to label groups of markers with genotypes that are similar to one another so that only one SNP from each group is used during filtering calculations. For *HapMap*, which had 36,638 markers, this resulted in 9,831 and 12,997 distinct labels for the *Major Filter* and the *Minor Filter*, respectively. For *WTCCC*, which had 13,943 markers, this resulted in 5,500 and 8,366 distinct labels for the *Major Filter* and the *Minor Filter*, respectively. This optimization reduces the considerable number of selected homozygous markers in filters. As a result, the running time for processing a query is reduced by half at the expense of higher candidate size. By using the optimization technique, the candidate fractions at the sensitivity of 99% and 98% are 20.1% and 11.1% higher than not using it. This technique was used in all experiments to expedite the computation; however using it remains optional as the difference in candidate size could be important in larger data sets.

**Figure 7:**
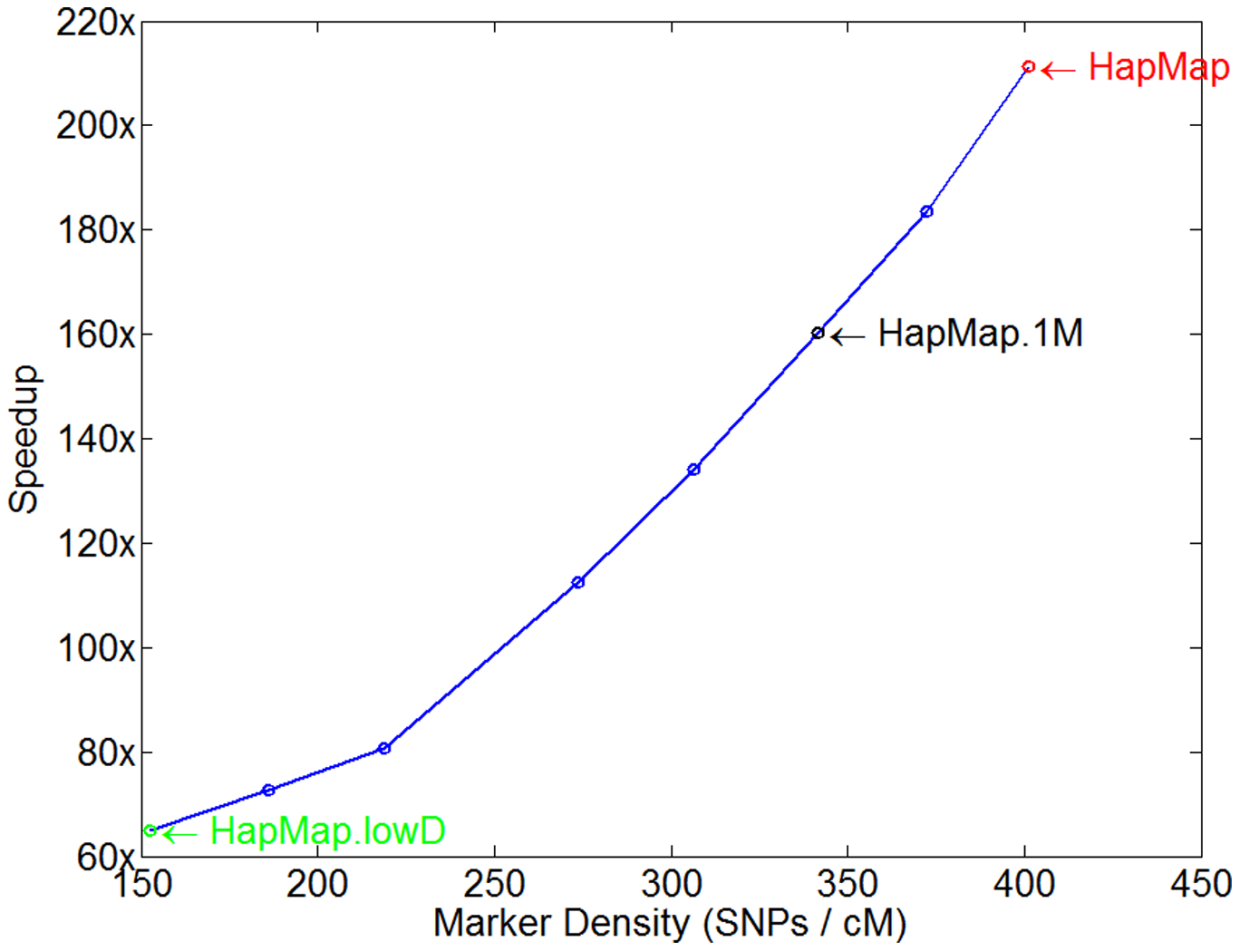
Performance vs. marker density. Markers were sub-sampled from the *HapMap* data set to varying degrees and speedup was measured with at a 99% sensitivity level. The densities corresponding to specific data sets used in other experiments are labeled.

Marker density has a strong effect on the performance of SpeeDB. We further explored their correlation by uniformly sub-sampling the markers in *HapMap* to various marker densities. The results shown in Fig. 7 are measured at a 99% sensitivity level. We observe that the speedup achieved by SpeeDB increases superlinearly with marker density, implying that SpeeDB can provide better pruning power if applied to data sets with high marker density. This promising result implies SpeeDB’s increasing relevance as researchers transition to using whole-genome sequencing instead of genotyping arrays.

**Figure 8:**
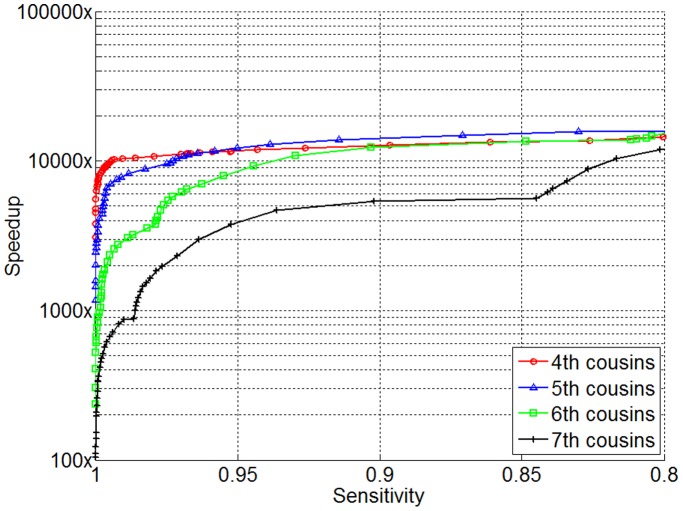
Performance of SpeeDB for identifying candidate close relatives. Performance was measured on query individuals that were simulated to have one IBD segment in size equal to 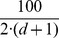 cM where 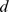 is the degree of cousin.

To investigate the utility of SpeeDB on identifying IBD between close relatives, which is important in hereditary disease studies, we simulated cousins of four different degrees based on the data in *WTCCC*. For each experiment, 10,000 queries were generated with each query simulated as a 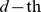 cousin of an indexed individual by injecting one IBD segment with length 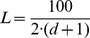 cM. 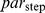 was set to 0.5 cM; 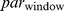 was set to the injected IBD segment size minus 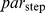, round up to closet half cM. The results on fourth to seventh cousins are presented in Fig. 8. Our results show that SpeeDB offers a very high pruning power when it is applied to detect close relatives, compared to the performance for detecting distant relatives sharing a 4 cM IBD segment. In particular, for fourth cousins as an example, SpeeDB achieves a 10,000x speedup with 99% sensitivity. Due to the fact that the number of selected homozygous markers within a window depends strongly on the window size, SpeeDB gradually loses its pruning power for more distant relatives. Nevertheless, even for seventh cousins, SpeeDB achieves a 867x speedup at a 99% sensitivity level.

SpeeDB is designed to provide a list of regions for each pair to downstream analysis methods. The algorithms used in GERMLINE, PARENTE, and fastIBD are amenable to using these regions to narrow the portion of the genome analyzed for each pair of individuals because they consider only local genomic information for IBD inference. This is on contrast to methods that consider the entire chromosome for each pair as is done with traditional HMM methods. The next version of PARENTE is being implemented to allow these regions to be specified and the implementations of GERMLINE and fastIBD can be modified in order to take these regions as input.

As variants detected through NGS data become increasingly available, the requirement for scalable analysis methods becomes clearer, and tools such as SpeeDB become an essential enabler for such large-scale studies. In particular, assuming millions of variants per samples and tens of millions unique variants across all individuals stored [17], we can extrapolate on the expected performance. For instance, when analyzing a database of 100,000 individuals, assuming 40 million genome-wide variants, and roughly 10% of them are non-reference in a query individual, if we index all the variants, the analysis would take a few hours. In reality, a large portion of variants have very low frequencies (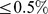). Because hardly any sample is homozygous minor at these markers, these data have almost no contribution to the pruning process. We can easily eliminate them from our analysis without sacrificing the performance. By doing so, we are able to improve pruning efficiency markedly.

## Conclusion

As studies grow to include larger cohorts, state-of-the-art IBD detection algorithms become computationally infeasible. To enable impending disease mapping studies of increasingly large cohorts, a scalable infrastructure is required. This infrastructure must be able to further support the ongoing nature of such studies by providing the ability to incorporate newly collected samples. We implemented SpeeDB to provide a practical and scalable infrastructure for such large-scale studies. Our method can be applied on unphased genotype data sets, tolerating genotyping error. We demonstrated the superior performance of SpeeDB on simulated individuals from Asian populations of the HapMap Phase III panel and the 1958 Birth Cohort of the WTCCC. As shown by our experiments, SpeeDB significantly reduced the examined candidate list by 200-fold for the case of 4cM IBD regions, further reducing the candidate set by 4 orders of magnitude for the common case of more recent common ancestors. The source code for SpeeDB is publicly and freely available at http://speedb.stanford.edu.

## Supporting Information

Figure S1 **The construction of composite haplotypes.**
(TIF)

Figure S2
**Varying two error tolerance thresholds** *p*_th_ **and** 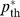 **results in a series of ROC curves.**
(TIF)

Figure S3 **Performance of SpeeDB for identifying 4 cM candidate IBD segments in** ***WTCCC.largeSet*** **.**
(TIF)
