# Supplementary figures and images for: An Effective Filter for IBD Detection in Large Data Sets
Source: PLoS One. 2014 Mar 25;9(3):e92713. doi: 10.1371/journal.pone.0092713 (PMC3965454; doi:10.1371/journal.pone.0092713)

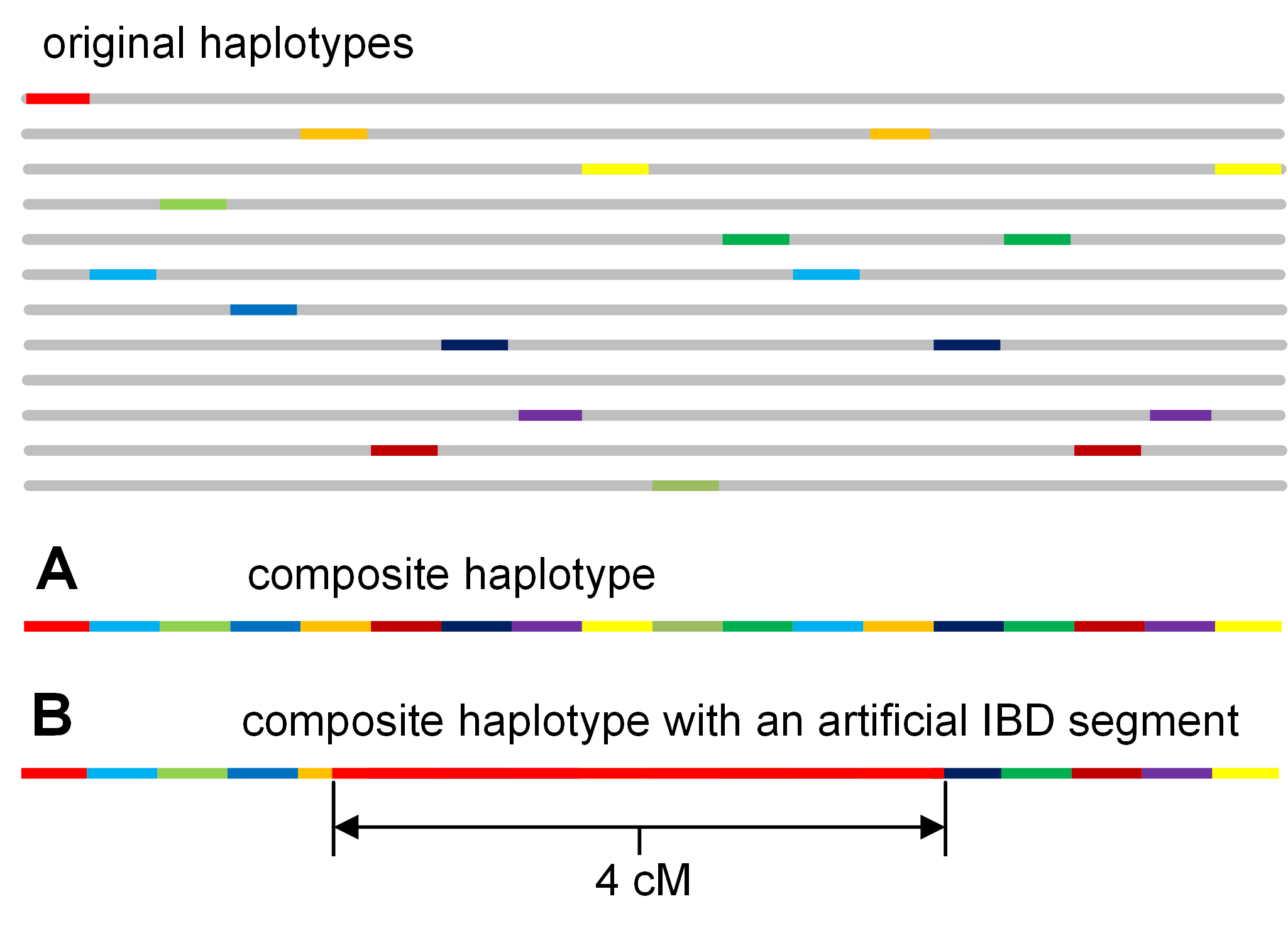

Supplement: Figure S1 — The construction of composite haplotypes. (TIF) [file pone.0092713.s001.tif]

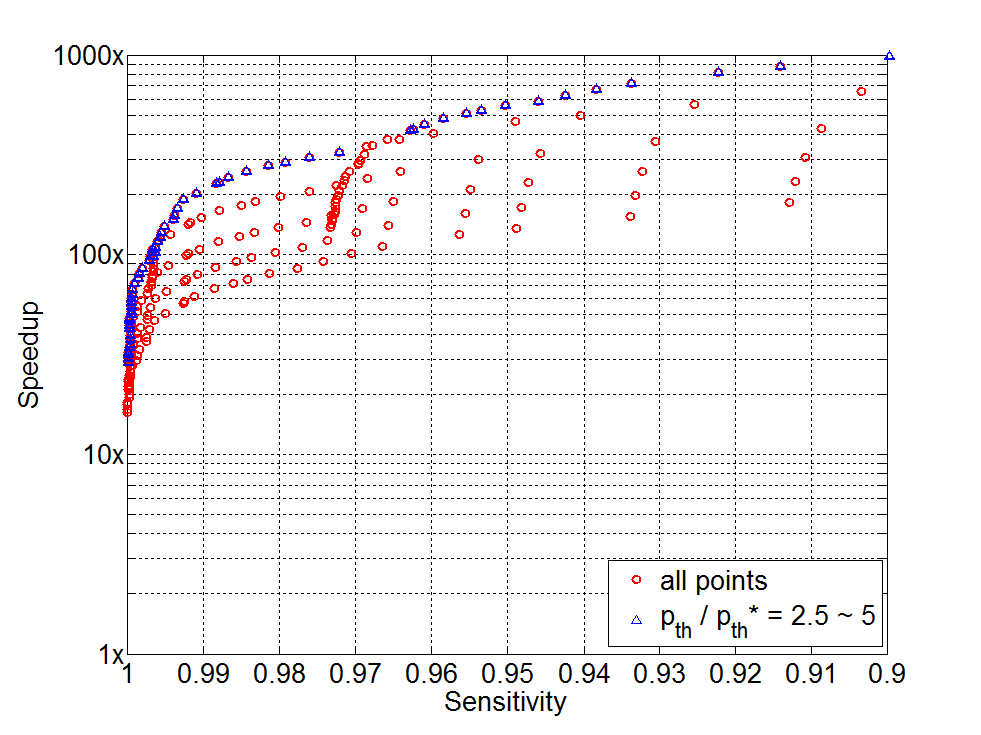

Supplement: Figure S2 — Varying two error tolerance thresholds p th and results in a series of ROC curves. (TIF) [file pone.0092713.s002.tif]

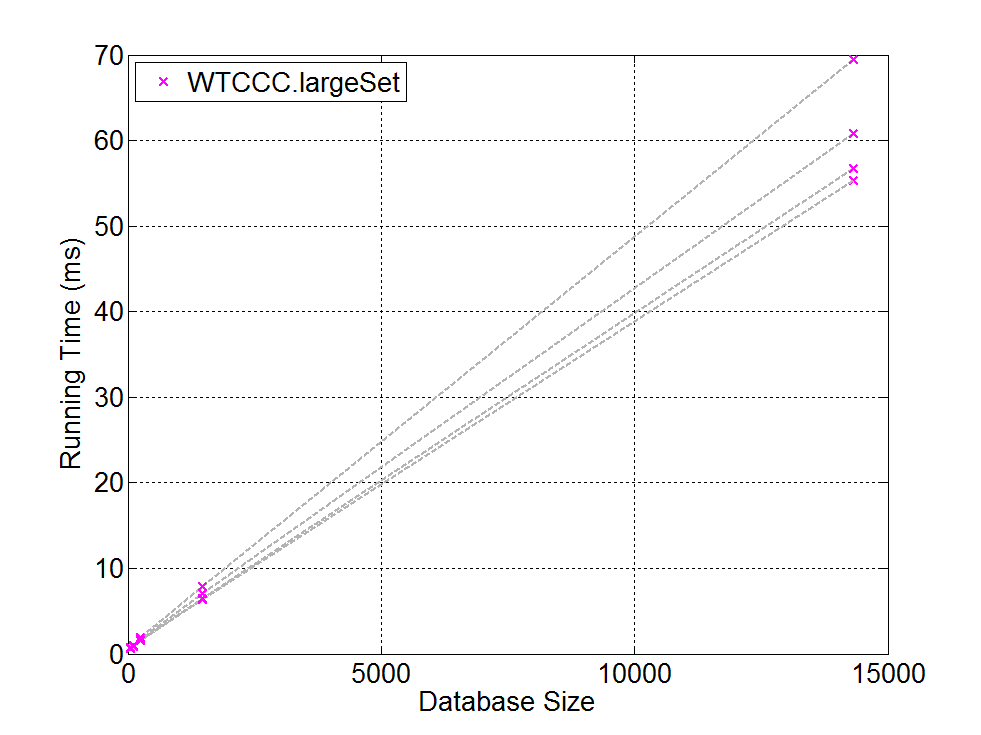

Supplement: Figure S3 — Performance of SpeeDB for identifying 4 cM candidate IBD segments in WTCCC.largeSet . (TIF) [file pone.0092713.s003.tif]
